# Supplementary material for: High‐Content SRS Imaging Unveils Altered Cholesterol Metabolism in Ovarian Cancers Under CAR‐T Treatment
Source: Adv Sci (Weinh). 2026 Feb 15;13(23):e18334. doi: 10.1002/advs.202518334 (PMC13104127; doi:10.1002/advs.202518334)
Supplement: Supplementary file 1 — Supporting file: advs74403‐sup‐0001‐SuppMat.docx. [file ADVS-13-e18334-s001.docx]

Supporting Information

High-content SRS imaging unveils altered cholesterol metabolism in ovarian cancers under CAR-T treatment

Chinmayee V. Prabhu Dessai, Zhuoying Huang, Haonan Lin, Guangrui Ding, Hongjian He, Menna Siddiqui, Meng Zhang, Wilson Wong*, Ji-Xin Cheng*


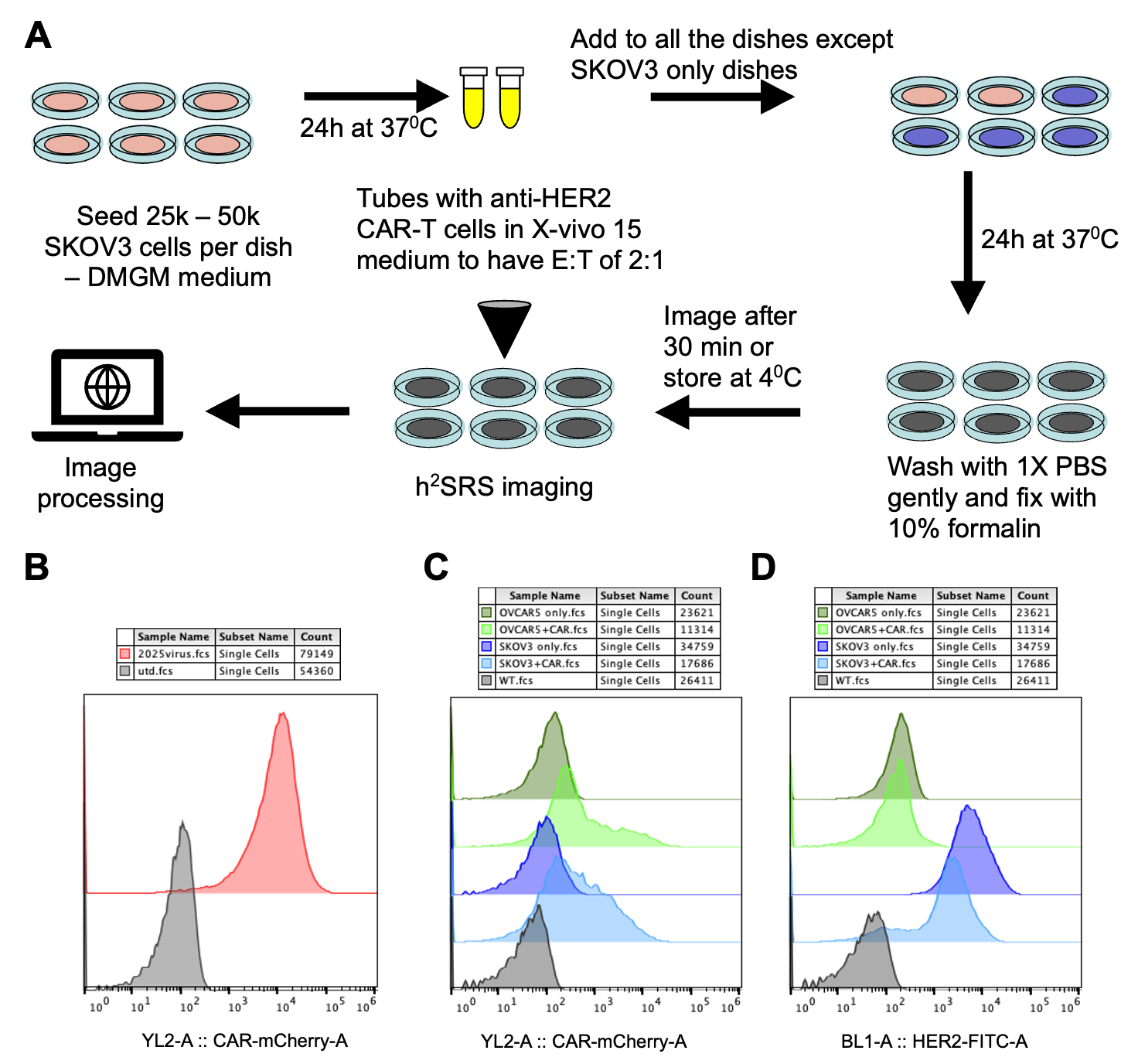


**Figure S1. Coculture and CAR and HER2 expression.** **(A)** Coculture sample preparation for hSRS. **(B)** CAR-expression in SKOV3 and Ovcar5 cells only and coculture with CAR-T cells **(C)** HER2 expression in SKOV3 and Ovcar5 with and without coculture with CAR-T cells


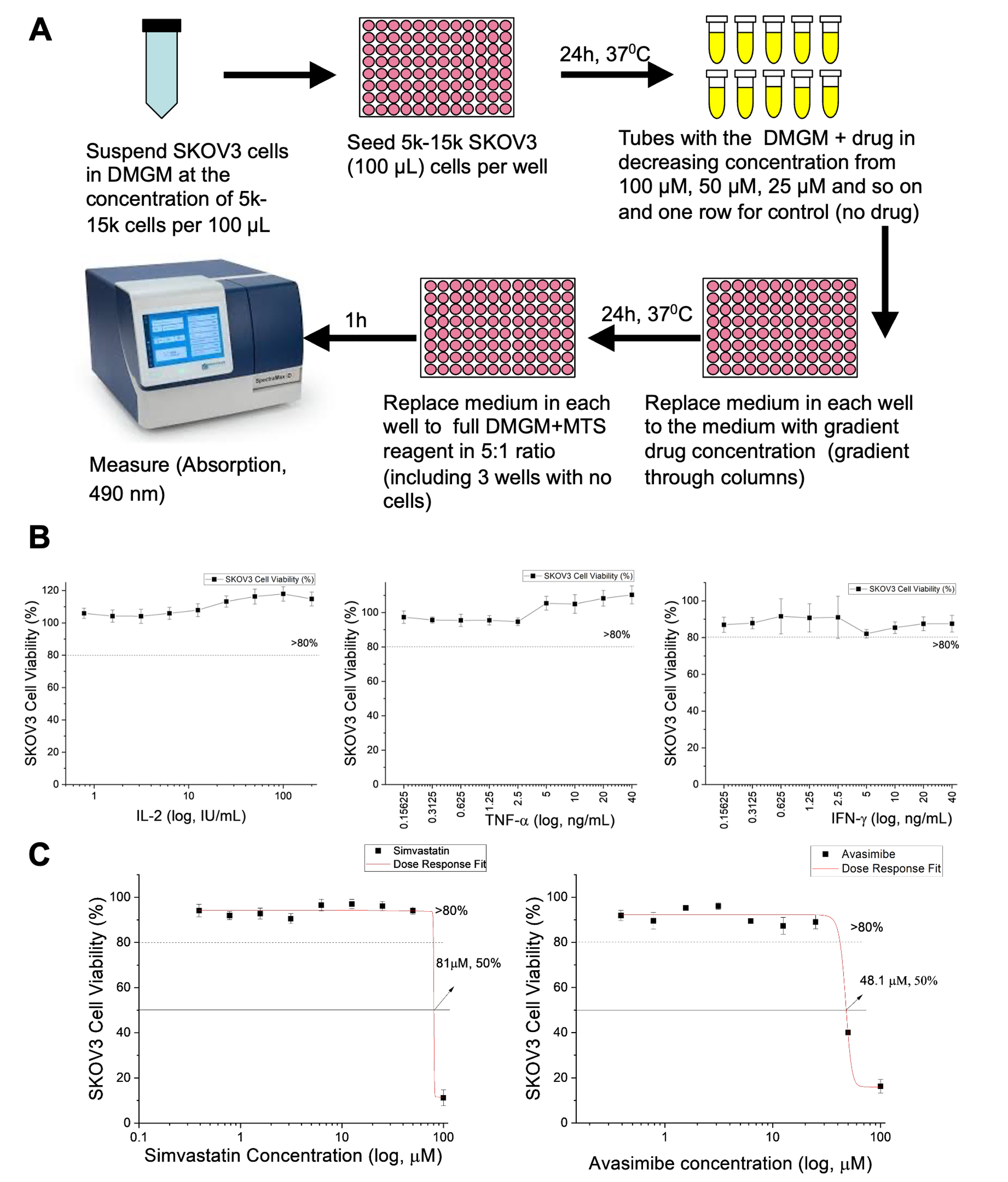


**Figure S2. Cell viability assay protocol and dose response curves. (A)** Cell Viability Assay protocol with MTS Reagent. **(B)** Cell dose response for pro-inflammatory cytokines: IL-2, TNF-𝛼, IFN-𝛾 on SKOV3 cells, n=6 for each curve. **(C)** Cell dose response for Avasimibe and Simvastatin on SKOV3 cells, n=6 for each curve.

**
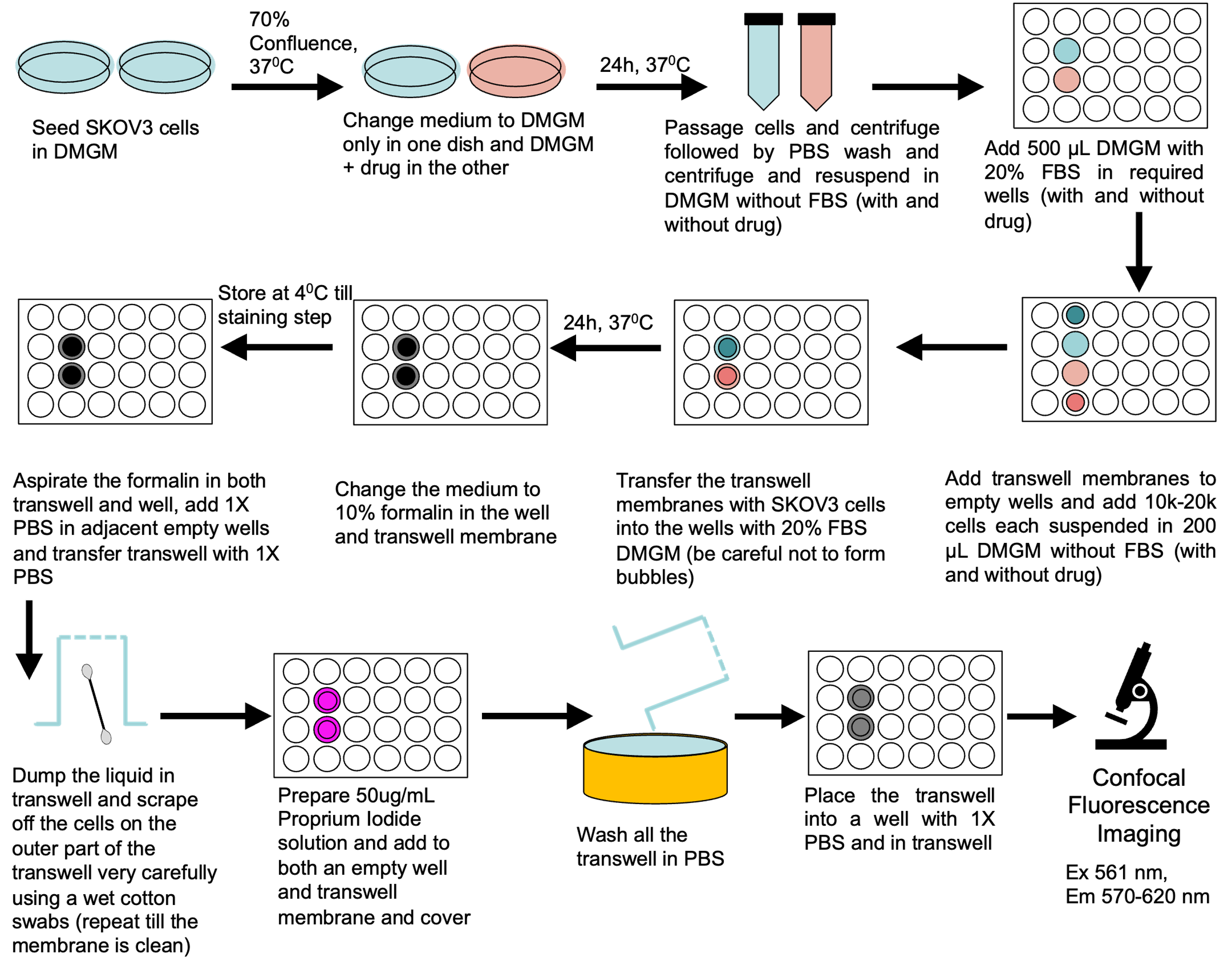
**

**Figure S3. Migration Assay.** Migration assay protocol with and without drug treatment.


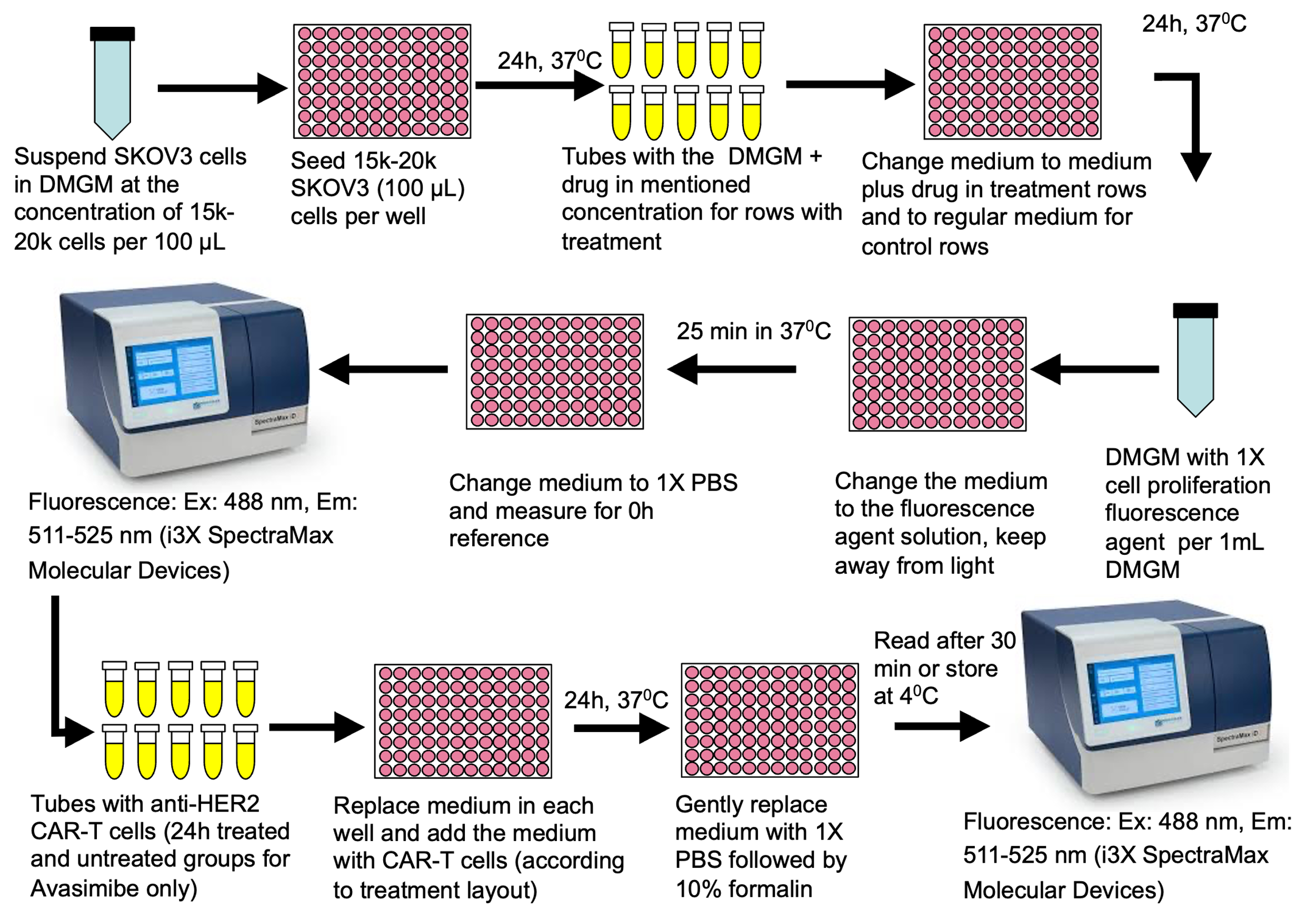


**Figure S4. Cytotoxicity Assay.** CAR-T cell induced cytotoxicity assay protocol in 2D cultures.


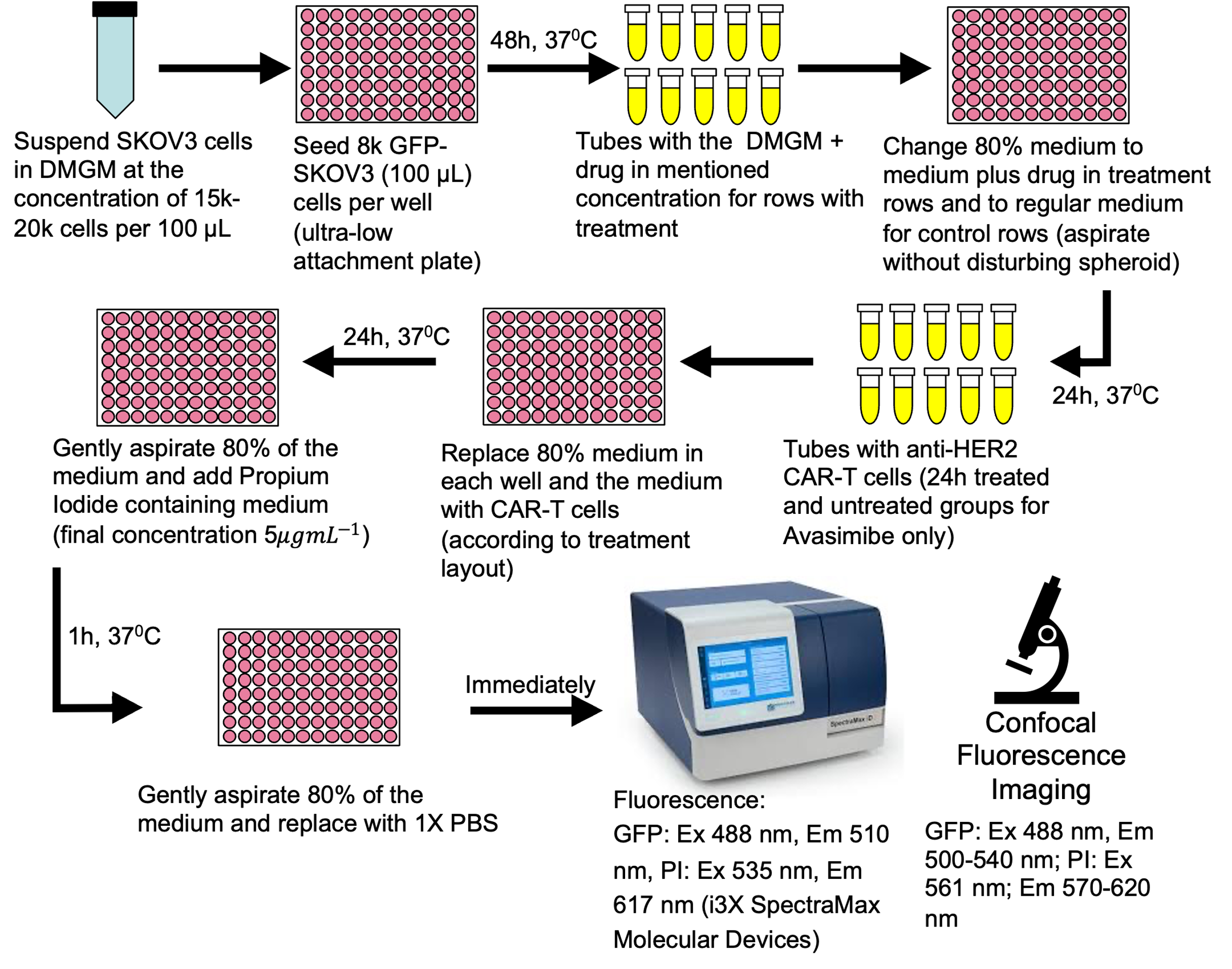


**Figure S5. 3D spheroids cytotoxicity Assay.** CAR-T cell induced cytotoxicity assay protocol in 3D cancer spheroids.


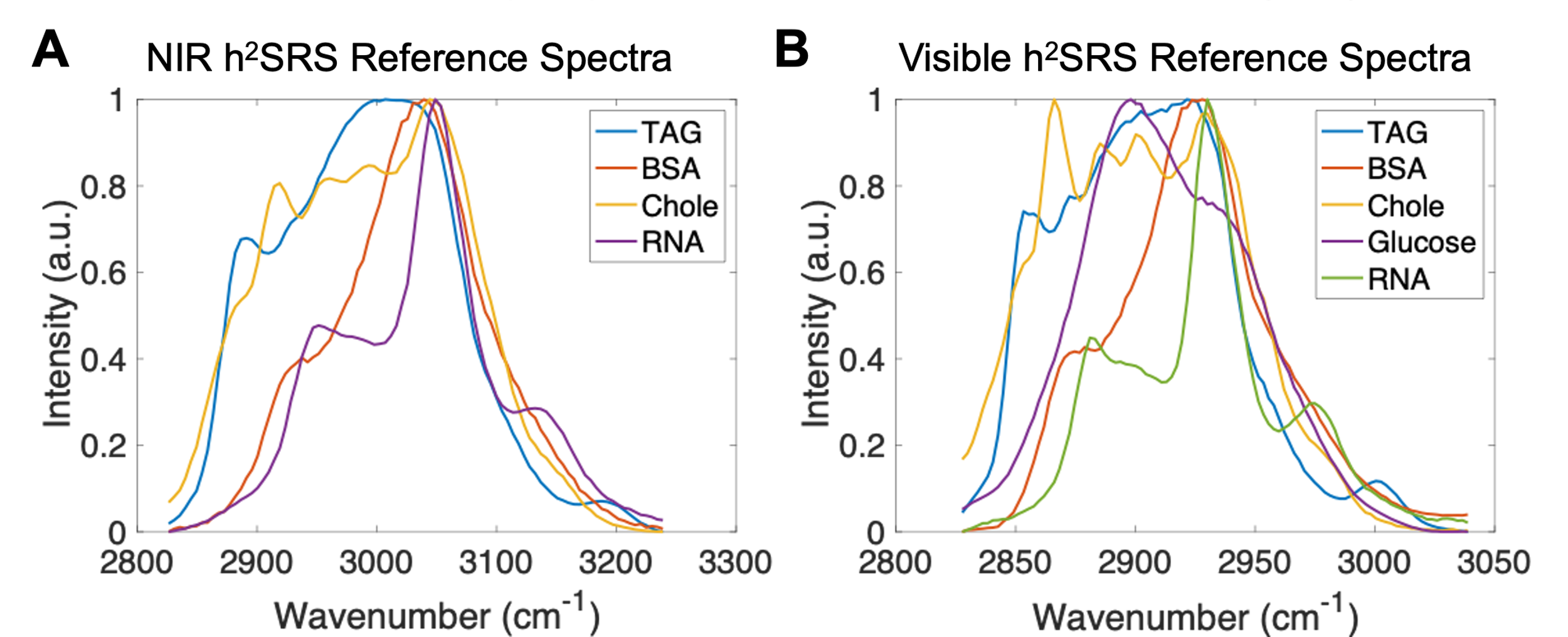


**Figure S6. Pure Chemical Spectra Used for input in h^2^SRS. (A)** NIR h^2^SRS Reference Spectra, **(B)** Visible h^2^SRS Reference Spectra


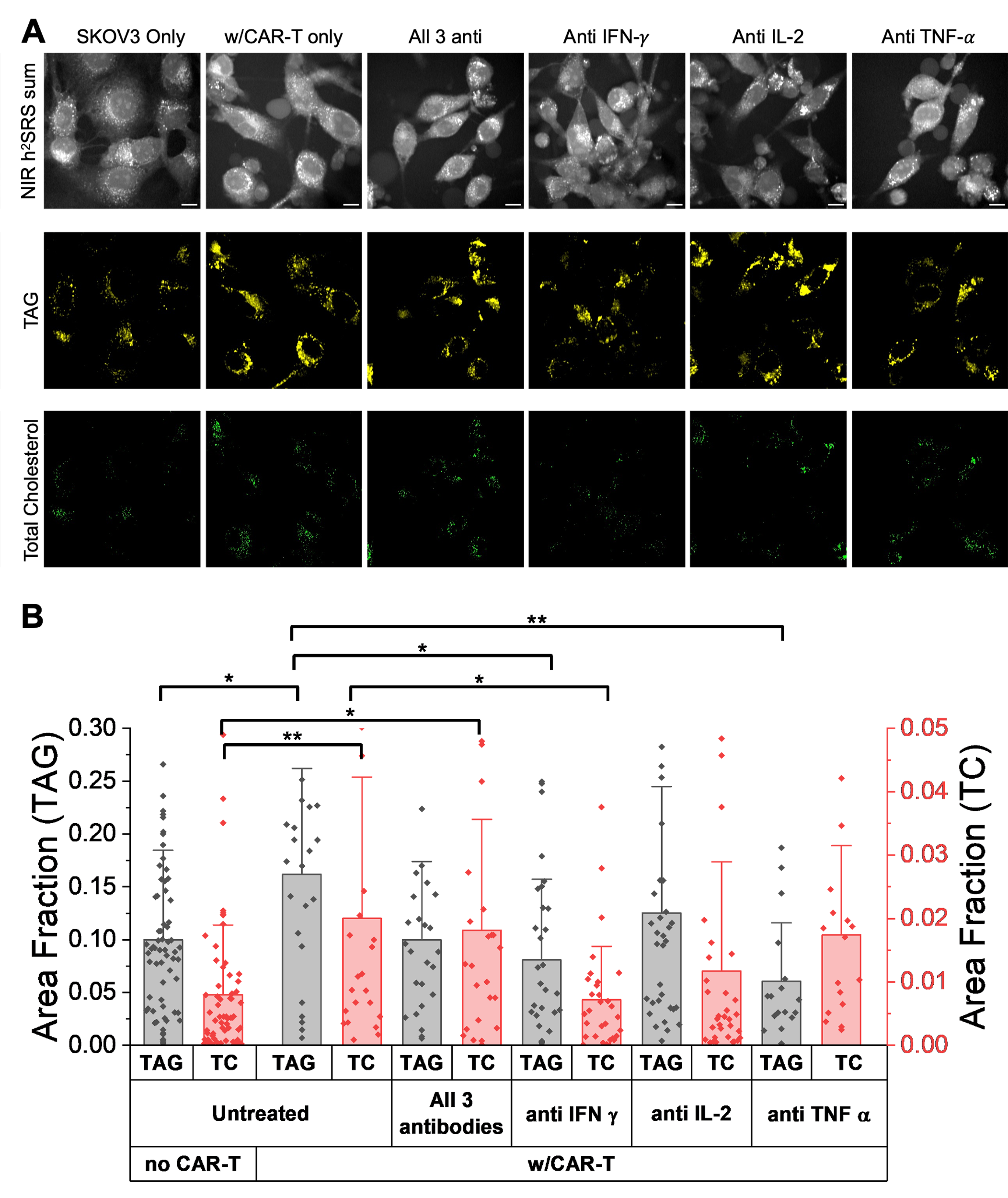
**Figure S7. Impact of cytokine neutralising on metabolism of SKOV3 cells under CAR-T challenge. (A)** TAG and TC NIR h^2^SRS imaging maps of SKOV3 cells with and without cytokine neutralising antibodies: anti-IL-2, , anti-IFN-𝛾, anti-TNF-𝛼, all 3 antibodies anti-IL-2 Concentration of each antibody: 5 $\mu$g/mL, scale bars: 10 μm. **(B)** Quantification of cellular TC and TAG in SKOV3 cells incubated with medium with and without cytokine neutralising antibodies cocultured with CAR-T cells, n>17 per group.


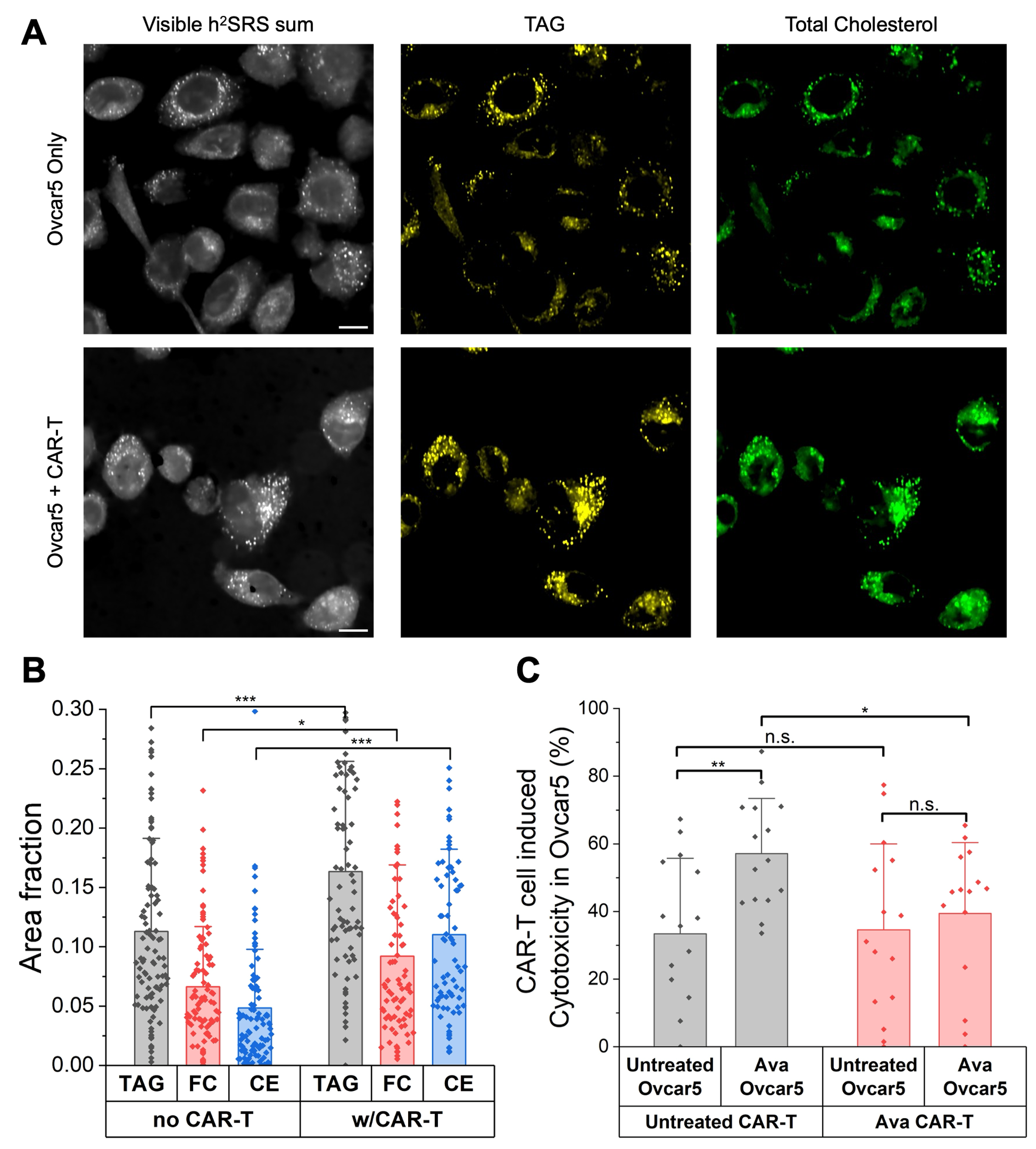


**Figure S8. Impact of CAR-T challenge on metabolism of Ovcar5 cells and Avasimibe treatment on CAR-T induced Cytotoxicity in Ovcar5 cells. (A)** Visible h^2^SRS imaging of the SKOV3 cells with and without CAR-T coculture, E:T = 2:1, scale bars: 10 μm. **(B)** Quantification of cellular FC, CE and TAG in Ovcar5 cells with and without coculture with CAR-T cells, n>22 per group. **(C)** Cell killing assay of untreated and Avasimibe treated Ovcar5 cells with untreated and Avasimibe treated CAR-T cells, n=15 per group, Avasimibe concentration: 30 μM for SKOV3 cells and 1 μM for CAR-T cells.


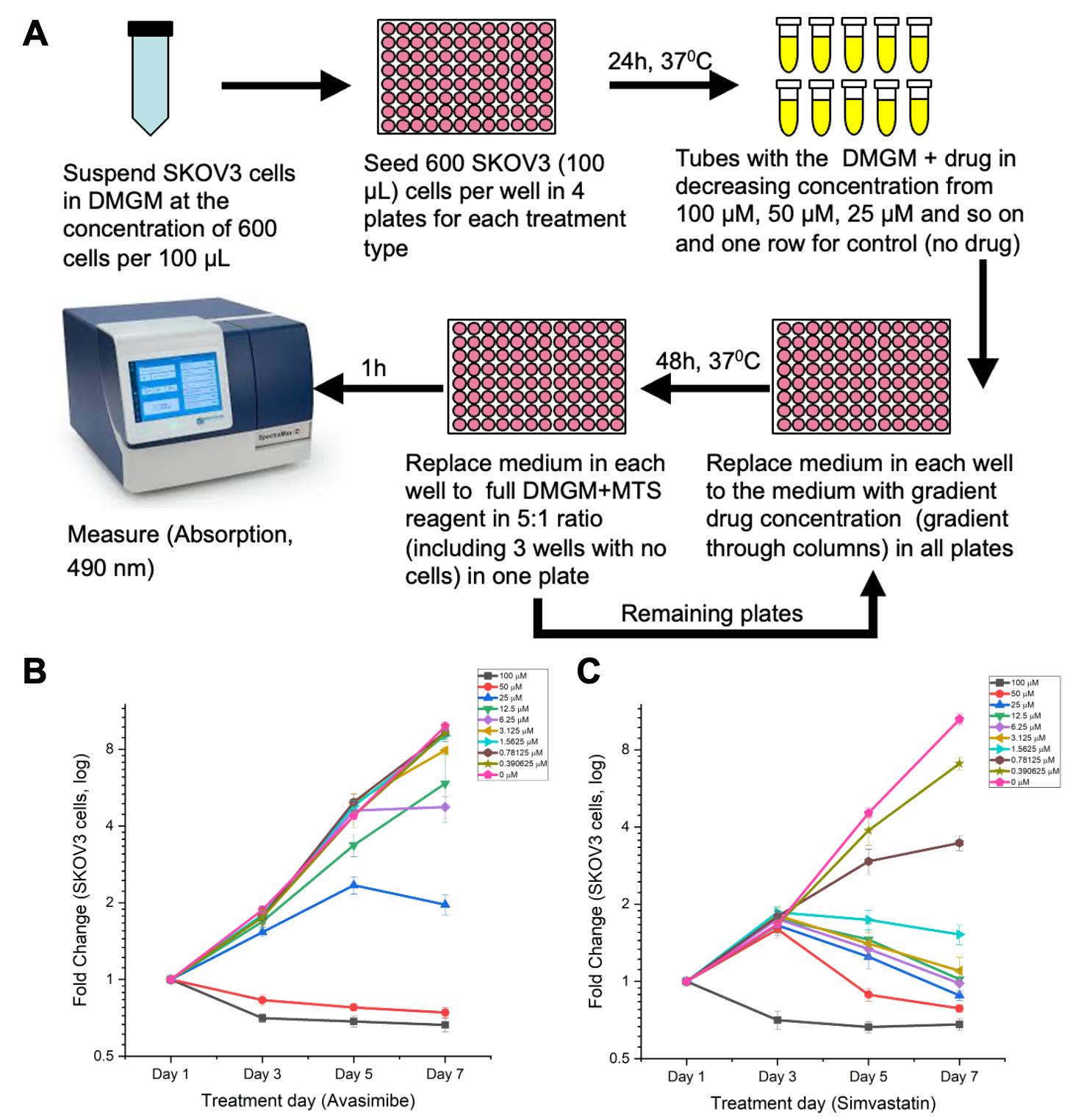


**Figure S9. Cell proliferation assay for SKOV3 cells. (A)** Cell proliferation assay protocol. **(B)** Cell proliferation assay with SKOV3 cells treated at varied concentrations of Avasimibe. **(C)** Cell proliferation assay with SKOV3 cells treated at varied concentrations of Simvastatin.

**
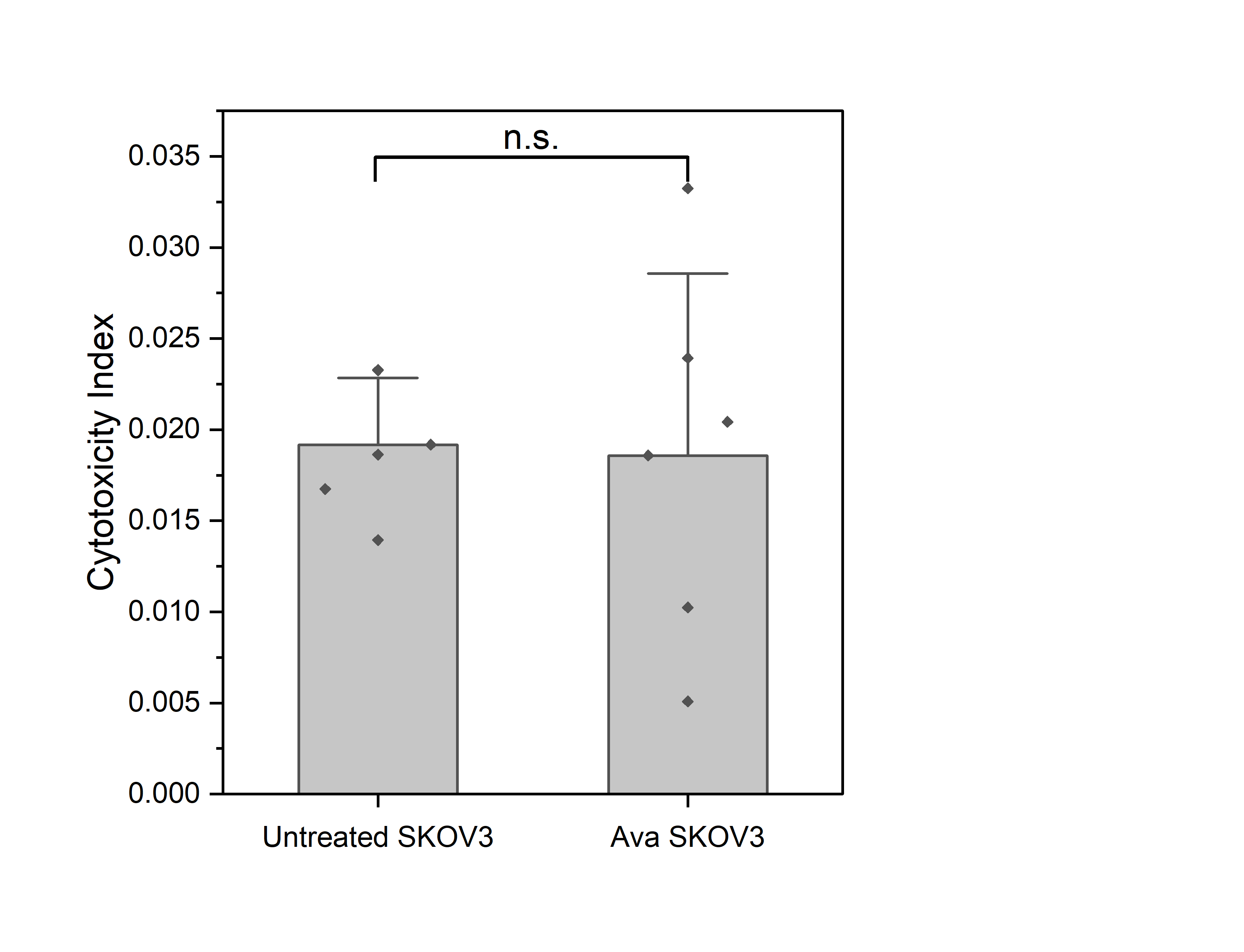
**

**Figure S10. 3D SKOV3 spheroids viability with and without Avasimibe treatment.** Avasimibe concentration: 30 μM.
